# The detrimental effects of progression of retinal degeneration in the visual cortex

**DOI:** 10.3389/fncel.2022.904175

**Published:** 2022-07-29

**Authors:** Anwesha Bhattacharyya

**Affiliations:** Amity Institute of Neuropsychology and Neurosciences, Amity University, Noida, UP, India

**Keywords:** primary visual cortex, retina, retinal degeneration, electrophysiology, morphology, rodents, humans

## Abstract

The leading cause of blindness in inherited and age-related retinal degeneration (RD) is the death of retinal photoreceptors such as rods and cones. The most prevalent form of RD is age-related macular degeneration (AMD) which affects the macula resulting in an irreversible loss of vision. The other is a heterogenous group of inherited disorders known as Retinitis Pigmentosa (RP) caused by the progressive loss of photoreceptors. Several approaches have been developed in recent years to artificially stimulate the remaining retinal neurons using optogenetics, retinal prostheses, and chemical photoswitches. However, the outcome of these strategies has been limited. The success of these treatments relies on the morphology, physiology, and proper functioning of the remaining intact structures in the downstream visual pathway. It is not completely understood what all alterations occur in the visual cortex during RD. In this review, I will discuss the known information in the literature about morphological and functional changes that occur in the visual cortex in rodents and humans during RD. The aim is to highlight the changes in the visual cortex that will be helpful for developing tools and strategies directed toward the restoration of high-resolution vision in patients with visual impairment.

## Significance statement

To achieve successful therapeutic intervention in RD, the structures downstream of the retina (lateral geniculate nucleus, LGN; and primary visual cortex, V1) should remain intact. Long-term sensory deprivation resulting from degenerating retina is associated with potential changes in the cortical circuit posing significant constraints on the success of RD treatment. Therefore, it is important to understand the consequences of RD on the state of the visual cortex for the successful implementation of restorative techniques. This review provides an up-to-date overview of the morphological and physiological changes in the visual cortex from various rodent and human studies.

## Introduction

The retina is a light-sensitive tissue layer at the back of the eye equipped with the necessary machinery to process light information to create an informative image of the external world. During retinal degeneration (RD) the retina undergoes deterioration with the consequent death of photoreceptors such as rods and cones. The progressive loss of receptors and loss of vision has a greater impact on daily life such as not being able to recognize faces, read and find objects. The most common form of RD is age-related macular degeneration (AMD) with vision loss in the elderly population. A recent report on numerous population-based studies indicates the emerging global burden arising with the increase in the number of people suffering from AMD from 196 million in 2020 to 288 million in 2040 (Wong et al., 2014). Another cause of RD is Retinitis Pigmentosa resulting from progressive loss of photoreceptors and the reginal pigment epithelium. An estimated 1.5 million individuals are affected with RP in the age group of 20–64 years (Bovolenta and Cisneros, 2009; Moore et al., 2020). Visual impairment not only has a personal impact but also represents a significant societal and economic burden. It thus poses a major health problem with an urgent medical need.

Several therapeutic strategies have been developed to combat RD, arising hope for patients. Such strategies include stem cell therapy (Ramsden et al., 2013), retinal prostheses (Shim et al., 2020), light-activated ion pump (Busskamp et al., 2010), photoswitch approaches modulating intrinsic channel (Polosukhina et al., 2012), and optogenetic gene therapy (van Wyk et al., 2015; Sahel et al., 2021). However, until now, all of them have had limitations in achieving the high-resolution vision in pre-clinical models as well as in patients. The success of these therapies relies heavily on the integrity of the visual system along the downstream region of the visual pathway (Kien et al., 2012).

There is abundant information about the progressive remodeling happening in the inner retinal neurons and glial cells following the death of photoreceptors (Jones et al., 1995; Marc et al., 2003; Anderson et al., 2016). Severe remodeling gradually affects the retinal ganglion cells (RGCs) at later stages (Garcia-Ayuso et al., 2019). On contrary, one finding shows that the dendritic structures of the RGCs remain intact and maintain stability even into the late stages of RD (Damiani et al., 2012; Lin and Peng, 2013; Anderson et al., 2016). For successful visual restoration, it is important to understand how the structures downstream in the visual pathway change over time. This review highlights the work that has examined the morphological and physiological changes in the visual pathway during RD.

Murine and rat models are widely used for RD research and have been advantageous in assisting the development of new therapies (Chung, 2017). There are a wide variety of animal models that have genetic mutations having resemblances with humans (Chang et al., 2002). These models have been very useful in understanding morphological and physiological changes. In contrast, rodent models of AMD are limited as they lack anatomical macula, have a lower percentage of cones, and the disease is not caused by a single genetic defect unlike RP but by several genetic polymorphisms. Further adding to the complexity are the anatomical differences between the human retina and AMD models (Pennesi et al., 2012; Soundara Pandi et al., 2021). Until now there are no animal models that can entirely represent the complete disease phenotype of AMD.

## Morphological changes in the visual cortex during RD

During early postnatal development the visual cortex undergoes dynamic changes (Himmelhan et al., 2018). Mice with RD in postnatal ages, P3–P28 show the same expression of ontogenetic markers, such as Nestin, Doublecortin, MAP2, Parvalbumin, and NeuN similar to wild-type mice. These proteins are expressed at different stages of development and some of them continue their expression until adulthood. Results demonstrate that these markers do not show any obvious deviation in cortical development during RD (Himmelhan et al., 2018). Until now, it is not completely clear if the visual cortex remains intact or undergoes structural changes following defects at the level of the retina. Humans suffering from MD undergo loss of vision in the central visual field. People who have an early onset of MD known as Juvenile Macular Degeneration (JMD) have different morphological changes compared to AMD. In AMD, only the structures downstream to the lateral geniculate bodies and frontal lobe white matter are affected while in JMD all the structures in the entire visual pathway (optic nerve, chiasm, lateral geniculate bodies, visual cortex) undergo volumetric reduction (Nuzzi et al., 2020). Additionally, AMD patients also have a reduction in the gray matter density following prolonged visual deprivation (Boucard et al., 2009). During MD the corresponding regions of the primary visual cortex (V1) deprived of retinal input result in the formation of silent cortical zones termed lesion projection zone (LPZ) (Ferreira et al., 2017). There is a discrepancy in the existing literature on whether new anatomical connections are generated with changes in the strength of the existing connections (plastic reorganization) with time (Wandell and Smirnakis, 2009). The impact of MD within the LPZ was assessed in a primate study and humans to see if the visual cortex undergoes reorganization by remapping visual inputs. The effects were assessed in a macaque monkey suffering from chronic MD (Shao et al., 2013). There was no significant change in the extent of the LPZ border and the deafferented region of V1. Consistently, the secondary visual area, V2 also had a similar trend with a stable LPZ border. However, it was interesting to note that area V5/MT showed significant activation suggesting marked reorganization. This could arise from the population receptive fields of the deafferented region of V5 that attain the capacity to be modulated by the visual stimulus presented outside the area of the retinal lesion. However, with due course of disease progression, these ectopic population receptive fields do not return to their original size indicating pronounced reorganization. Similar results were also obtained from AMD and JMD individuals where remapping was largely absent in the early visual areas (Baseler et al., 2011). These results corroborate the fact that there is limited reorganization in the early visual areas due to the absence of significant visual modulation despite the loss of vision.

It has been shown that the visual white matter pathway is greatly affected in age-related diseases as well as in several eye diseases (Wang et al., 2012). The white matter tissue organization within the optic radiation in AMD subjects has profound damage correlating with the loss in visual acuity (Yoshimine et al., 2018). Another form of AMD such as the neovascular form of AMD has an acute onset with leakage of fluid and hemorrhage in the central macula and these patients have different short- and long-term changes in the visual cortex (Hanson et al., 2019). The patients affected show no changes in the cortical volume of the occipital lobe of the brain within 3 months of disease diagnosis. However, these patients showed a decrease in cortical volume after long-term assessment (~5 years) in the LPZ region of the visual cortex. The central cortical region of V1 deprived of visual input also undergoes significant thinning, unlike the peripherally responsive area that becomes thicker as a compensatory mechanism of improvement in peripheral vision (Burge et al., 2016).

In RP, the degeneration starts in the periphery and advances toward the central retina. These patients display no changes in the thickness of gray matter but undergo a shift of central retinal representation to further peripheral locations (Ferreira et al., 2017). One plausible reason for the remapping could be rapid adaptation following degeneration. The neurons from the central visual field exert influence on the deafferented peripheral visual field by strengthening the long-range horizontal connections within V1. The retinotopic changes thus help in the stabilization of central vision increasing the necessity to facilitate visual plasticity. In addition, there are changes in the anatomical connectivity across the visual areas V1–V3 (Fine and Park, 2018). In the absence of visual input, the occipital cortex is recruited for various auditory and tactile tasks resulting in cross-modal plasticity (Cunningham et al., 2015). The human medial temporal complex (hMT+) present in the dorsal visual stream gets tuned to perform comparable computations as in the visual cortex. The corpus callosum which is a channel of information processing between two hemispheres is induced by plastic rearrangements due to visual dysfunction.

The V1 and the association cortices undergo significant changes in the gray matter volume restricted to the occipital cortex of patients with RP. The extent of loss is associated with the extent of peripheral visual degeneration (Rita Machado et al., 2017). The differences observed in spatial reorganization in RP and AMD patients are most likely due to distinct processing of central and peripheral vision, and different connections of the central and peripheral visual neurons with subcortical and higher-order cortical areas. Another reason could be the death of the RGCs leading to volumetric reduction of the visual cortex weakening the chances of functional reorganization in AMD patients (Hernowo et al., 2014). The summary of the morphological changes in the downstream visual pathway during RD in humans and rodents is listed in Table 1. These studies indicate that the changes in the visual cortex following RD may not be apparent initially after disease onset but may emerge slowly over time resulting from prolonged loss of input.

**Table 1:** Summary of morphological studies of retinal degeneration effects on the visual cortex.

**Model**	**Study**	**Effects**	**References**
Humans (congenital blind patients; (age: 19–47 years)	Morphological changes in visual pathways (optic nerve, tract, radiations and occipital cortex using magnetic resonance imaging (MRI)	Structural changes such as “thinning” or atrophy of the visual pathways (optic nerve, chiasm and tract). No effect was observed in the visual occipital cortex	Breitenseher et al., 1998 Neuroradiology
Humans (AMD patients age; 51–82 years)	Changes in gray matter density associated with prolonged sensory deprivation in visual cortex using magnetic resonance imaging (MRI)	Reduction of gray matter density in visual cortex (posterior part of calcarine fissure)	Boucard et al., 2009 Brain
Humans (JMD patients)	Structural changes in the central nervous system in patients suffering from JMD	Volumetric reduction of white matter along the entire retinotopic visual pathway (optic nerve, chiasm, lateral geniculate bodies, optic radiation and visual cortex). Reduction of gray matter density in the posterior part of calcarine fissure in the occipital pole	Nuzzi and Vitale, 2021 Eye and Brain Nuzzi et al., 2020 Frontiers in Neuroscience
Humans (patients with RP; age: 20–66 years)	Primary visual cortical retinotopic remapping and thickness of V1 using MRI	The central retinal representations shifted to more peripheral locations dependent on the extent of visual loss. No structural changes were observed in the V1	Ferreira et al., 2017 Neuroimage
Humans (patients with RP having low partially preserved vision; age: 20–66 years)	Effects of peripheral visual loss on the gray matter volume of the occipital cortex	Reduction in gray matter volume in V1, calcarine sulcus, lingual gyri, cuneus, right occipital superior gyrus. The loss was associated with the magnitude of peripheral visual degeneration.	Rita Machado et al., 2017 Scientific Reports
Humans (AMD patients; age:62–84 years)	Changes in white matter fascicles within optic radiation that project to primary visual cortex (V1)	Damage in the white matter tissue of optic radiation (OR) fascicles projecting to V1 correlating to the extent of visual impairment	Yoshimine et al., 2018 Brain Structure and Function
C3H/HeNRj rd mouse (P3–P40)	Effects of RD on visual cortex formation during early postnatal development	Cortex formation is normal and doesn't undergo any alterations during development	Himmelhan et al., 2018, Mechanisms of development
Human (patients with acute unilateral neovascular nvAMD; age 67–81 years baseline, 73–86 years for follow up)	Assess time course of structural changes in the visual cortex during substantial loss of unilateral visual input in nvAMD.	Cortical atrophy was detected in a period of several years in the macular LPZ devoid of long-standing retinal input	Hanson et al., 2019 IOVS

## Physiological changes in the visual cortex during RD

### Animal studies

In a rat model of retinal dystrophy (RCS rat), the extent of pattern-vision degeneration was demonstrated using a short pulse of bright white light and gratings of different spatial frequencies (patterns) (Gias et al., 2011). Analysis of both multiunit activity (MUA) and local field potentials (LFPs) showed a marked decrease in responses and increased latency across different layers of the visual cortex. The degenerated group had reduced responses across different spatial frequencies and the observations were pronounced as early as 4 weeks of age. This demonstrates that conscious perception can get affected even at the very early stages of RD, i.e., within the first postnatal months.

Another study in a transgenic rat model of RD, S334ter-3 rats, aged between 2 and 3 months (has a rhodopsin mutation like human RP patients) evaluated the changes in the electrophysiological properties of the V1 such as orientation selectivity, spatial, and temporal frequency tuning and receptive field size. Degenerated rats had diminished orientation selectivity mostly in the lower layers of the cortex (layers V-VI) with better responses only at lower spatial and temporal frequencies. The size of the receptive field was smaller compared to normal seeing rats (Chen et al., 2016). Recently, Chen and colleagues have done an elaborate study to examine how the contrast response properties in V1 neurons are affected by degeneration. The contrast response function was analyzed for each cell where higher spontaneous activity was observed in absence of contrast and weak stimulus-evoked activity at both medium and higher contrasts (30 and 100%). The firing rate was low across three different contrast levels. It is surprising to note that the contrast sensitivity (CS) semi-saturation contrast (C_50_) did not show any significant alterations in response to spatial-temporal variations in luminance contrast change. Overall these results indicate the diminished capacity of discriminating stimuli under different contrast conditions and adaptation of the visual system to environmental contrast (Chen et al., 2020).

Spontaneous activity is essential in establishing appropriate connectivity during the early development of the visual circuit and maintenance of topographic maps (Tritsch et al., 2007; Wosniack et al., 2021). The V1 of S334ter rats demonstrates an increase in spontaneous activity and a reduced capacity to elicit responses generated by novel stimuli (Wang et al., 2016). The elevated activity could be due to morphological changes in the visual pathway and altered function of the V1 (Hubel and Wiesel, 1964). Despite this, the visual cortex has a remarkable capacity of undergoing plastic changes even at later stages of degeneration to improve the signal quality of degraded input (Begenisic et al., 2020). As a compensatory mechanism excitation/inhibition (E/I) balance in the V1 is altered following an increase in the inhibitory drive thus improving the signal-to-noise ratio (Pietra et al., 2021). This could be due to the activation of parvalbumin-containing Gamma-aminobutyric acid (GABA) neurons that inhibit pyramidal cell activity (Bhattacharyya et al., 2013).

To gain better insights into the cortical effects of RD, neural activity in response to light flashes of different intensity were measured from V1. For this mutual information (MI) was used as a measure to determine the efficacy of information transmission between visual stimulus and neuronal activity (Wang et al., 2018). The MI of spiking activity and LFPs were compared as a better predictor of how well the visual information can be decoded by the V1 neurons. This is also helpful to understand the ability of the cortex to respond under pathological conditions in the retina. There was a substantial decrease in firing rate compared to control, a variability of responses with different light intensities, and an overall decrease in MI of responsive activities. The LFP signals were able to encode the light intensity consistently and reliably across several trials, especially in the delta and beta bands. This emphasizes that the low-frequency activity is possibly a better predictor of information processing in the visual cortex during cortical reorganization. The work reviewed suggests that while vision is impaired there are remaining neurons in the visual pathway that retain the ability to capture visual information.

### Human studies

The signals from the retina are relayed to the primary visual cortex (V1) for processing visual information and conscious visual perception. During RD the retina undergoes a profound increase in spontaneous activity as a result of increased glutamate concentration, and altered synaptic input due to the death of photoreceptors (Marc et al., 2007). It is important to understand how these changes in the retina affect the functioning and information processing in the downstream visual pathway (Table 2). The loss of retinal input causes the visual cortex to undergo functional adaptations in its circuitry, which can have significant repercussions on the current therapeutic approaches. A summary of the salient effects in the visual cortex during RP and AMD disease conditions in humans is briefly summarized in Table 2.

**Table 2:** Summary of physiological studies of retinal degeneration effects on the visual cortex.

**Model**	**Study**	**Effects**	**References**
S334TER-3 Rats (age: 2–3 months of age)	Changes in electrophysiological properties in V1 such as orientation tuning, spatial and temporal frequency, receptive field (RF)	Weakening of orientation selectivity, reduced spatial and temporal frequency, decrease in receptive field size	Chen et al., 2016 Scientific reports
S334TER-3 Rats (age: 2–3 months of age)	Changes in spontaneous activity, firing rate, interspike interval (ISI) and Lempel-Ziv (LZ) complexity were analyzed	Increased firing rate, decrease in ISI and lower LZ complexity	Wang et al., 2016 Neuroscience Letters
S334TER-3 Rats (age: 2 months)	Efficiency of information transmission was quantified in V1 by determining the mutual information (MI)	Decrease in MI between visual stimulation and neural response in V1	Wang et al., 2017 IEEE
S334TER-3 Rats (age: 3–4 months)	Efficiency of information transmission was quantified in V1 by determining the mutual information (MI). The analysis was based on both spikes and local field potential (LFP) signals	Decrease in MI between visual stimulation and neural responses for spiking activity, whereas for LFP the MI was similar to control group implying the ability of the visual system to capture information at population level	Wang et al., 2018 Neuroscience
S334TER-3 Rats (Age: 3–4 months)	Evaluation of residual contrast response properties after retinal degeneration	The spontaneous activity was strong and evoked responses was weaker observed at medium and high contrast level. No significant difference in C_50_ (semi-saturation contrast) was observed	Chen et al., 2020 Vision Research
Humans (patients with RP; age: 23–57 years)	Evaluation of residual visual function by measuring pattern reversal evoked potential (PVEPs) using different sizes of black and white checks of different contrast levels	A decrease in amplitude (P100) and increase in latency at lower contrast but not at higher contrast.	Mancebo-Azor et al., 2020 MDPI
Royal College of Surgeons (RCS) rat; age- P (28–35), P (49–56), P (98–105)	Determine loss of pattern vision by measuring (a) optical imaging responses using grating stimulus (b) Multi unit activity (MUA) and Local field potentials (LFPs) were measured in response to light stimulus in different layers of cortex	A marked decrease in amplitude of cortical signals were observed in rats for a wide range of spatial frequencies and for all groups. The MUA responses showed a decrease in older age group with reduced firing rate. The amplitude and latency of the LPFs that decreased at early ages increased progressively with age. The effect was similar in all cortical layers.	Gias et al., 2011 Vision Research
rd10 mice Age: P60, P120, P180	Functional changes in the visual cortex circuit during progressive retinal degeneration	Significant alteration in the excitation/ inhibition balance resulting from increased inhibition in local circuits.	Pietra et al., 2021 Visual Neuroscience
Humans patients with inherited RD (age: 36-57 years)	Contrast sensitivity deficits in patients with inherited retinal degeneration	Patients with IRD exhibit significant deficits in contrast sensitivity despite having normal visual acuity. The contrast sensitivity was measured by a new method named as Quick Contrast Sensitivity Function (QCSF) that allows a more comprehensive evaluation using a greater number of contrast and spatial frequency evaluation.	Alahmadi et al., 2018 BMC Opththalmology

A lack of stimulation in the visual cortex due to a retinal lesion in AMD is characterized by a central scotoma, loss of central vision, and impaired visual acuity. The loss of central vision can result in the formation of a new point of focus through eccentric fixation called preferred retinal locus (PRL). Visual stimulation in these affected individuals shows decreased activity of the visual cortex and elevated activity in a few associative areas outside the visual field responsible for eye coordination such as frontal and supplementary eye fields, prefrontal cortex, intraparietal sulci, and parietal lobule (Alahmadi et al., 2018). Another finding has also shown that AMD patients are unable to process fine details of the daily visual scenes with high spatial frequency (Ramanoel et al., 2018). Increasing the contrast level enhances the cortical responses and improves the processing of high spatial frequencies in scenes. CS is an important aspect of visual function useful for providing information about defects in perception. Deficits in CS contribute extensively to poor visual function even when visual acuity is unaffected. Implementing new methods such as Quick Contrast Sensitivity Function (QCSF) provides a comprehensive evaluation of deterioration in CS (Alahmadi et al., 2018). This test can use a wide range of contrast and spatial frequency combinations. Patients diagnosed with genetic inherited RD exhibit visual deficits despite no significant alterations in their visual acuity. This arises due to significant decreases in the CS at all spatial frequencies with pronounced effects at higher spatial frequencies.

The effect of degeneration on contrast processing was also determined in patients with RP by measuring pattern reversal visual evoked potentials (PVEPs) (Mancebo-Azor et al., 2020). The patients had a reduced amplitude and increased latency of **P100 amplitude** for different sizes of checks at low contrasts (16% and 6%) but not at higher contrasts. The authors hypothesized that the observed differences between low and high contrast could be due to different processing streams (parvocellular and magnocellular) with different functional properties contributing to this effect. The results are in line with previous studies in rodents and corroborate the fact that degeneration affects the functioning of the cortex in a wider manner. Recent studies in rodents and humans show that the adult brain has the ability to undergo short-term plasticity to adapt to visual changes (Lunghi et al., 2019; Begenisic et al., 2020). This is promising since it can be advantageous for possible therapies to restore vision (Castaldi et al., 2016). Taken together, the above findings suggest that although morphological changes happen in the early and late stages of the visual pathway the capability of stimulus-dependent remodeling until advanced stages of degeneration can be beneficial in the development of visual prosthesis.

## Conclusion

Understanding the status of the visual cortex during the progressive death of the photoreceptors is essential for effective restorative techniques. The loss of photoreceptor input upon degeneration does not have any specific impact on the maturation and structure of the visual cortex but more generally affects the brain tissue properties. On the other hand, visual cortex neurons exhibit substantial changes in the electrophysiological properties during RD affecting their physiological activity. The retina undergoes profound morphological and functional changes resulting in reduced visual sensitivity that can potentially disrupt the efficiency of information processing in the visual cortex.

The preservation of the lower order neurons and the downstream retinal circuitry have encouraged scientists and clinicians to develop several therapeutic approaches such as retinal prosthetics and optogenetic gene therapy in restoring vision (McClements et al., 2020; Shim et al., 2020). Despite several advancements, the quality of the visual percept obtained after therapy is of relatively low quality and not enough to lead an independent life. One plausible reason for this could be the age at which therapeutic intervention is initiated. At advanced stages of degeneration when the rods are completely absent and with remnant cones, rescue strategies to obtain high acuity vision may be hindered due to disruption of excitation-inhibition balance. It is therefore necessary and significant to evaluate the changes in neural activities in the primary visual cortex during RD. Despite the fact that the rd1 mouse model of RD is widely used for optogenetic gene therapy and pre-clinical testing of inherited retinal disease, there is hardly any study evaluating changes in the visual cortex of these mice, most of the cortical work having been performed in rats. To make further advancements in pre-clinical testing it is essential to evaluate the cortical activity at different stages of RD facilitating the development of optogenetic gene therapy or visual prosthesis to achieve successful vision restoration.

## Author contributions

The author confirms being the sole contributor of this work and has approved it for publication.

## Funding

AB was supported by the Ramanujan Fellowship of Science and Engineering Research Board (SERB), Department of Science and Technology (DST), Govt. of India (Grant: RJF/2019/000040), and the Core Research Grant (CRG) of SERB, DST (CRG/2021/003472).

## Conflict of interest

The author declares that the research was conducted in the absence of any commercial or financial relationships that could be construed as a potential conflict of interest.

## Publisher's note

All claims expressed in this article are solely those of the authors and do not necessarily represent those of their affiliated organizations, or those of the publisher, the editors and the reviewers. Any product that may be evaluated in this article, or claim that may be made by its manufacturer, is not guaranteed or endorsed by the publisher.
